# Probing the coupling of a dipole-bound electron with a molecular core†
†Electronic supplementary information (ESI) available: The calculated wave function of the dipole-bound excited state of C_2_P^–^, more experimental details, a description of photoelectron angular distributions and the measured beta values. See DOI: 10.1039/c8sc04771e


**DOI:** 10.1039/c8sc04771e

**Published:** 2018-11-15

**Authors:** Joseph Czekner, Ling Fung Cheung, G. Stephen Kocheril, Lai-Sheng Wang

**Affiliations:** a Brown University , Department of Chemistry , 324 Brook Street , Providence , RI 02912 , USA . Email: lai-sheng_wang@brown.edu

## Abstract

A dipolar molecule can weakly bind an electron in a diffuse orbital. However, the spin–orbit coupling between this weakly bound electron and the electrons in the molecular core is not known. Here we probe this coupling using the linear C_2_P^–^ anion with the ^3^Σ^+^ ground state, which possesses dipole-bound excited states because neutral C_2_P (^2^Π) has a sufficiently large dipole moment. Photodetachment spectroscopy and resonant photoelectron spectroscopy are used to probe the nature of the dipole-bound states. Two dipole-bound excited states are observed with a binding energy of 37 cm^–1^, corresponding to the two spin–orbit states of neutral C_2_P (^2^Π_1/2_ and ^2^Π_3/2_). The current study demonstrates that the weakly bound electron in the dipole-bound excited states of C_2_P^–^ is not spin-coupled to the electrons in the C_2_P core and can be considered as a quasi-free electron.

## Abstract 2

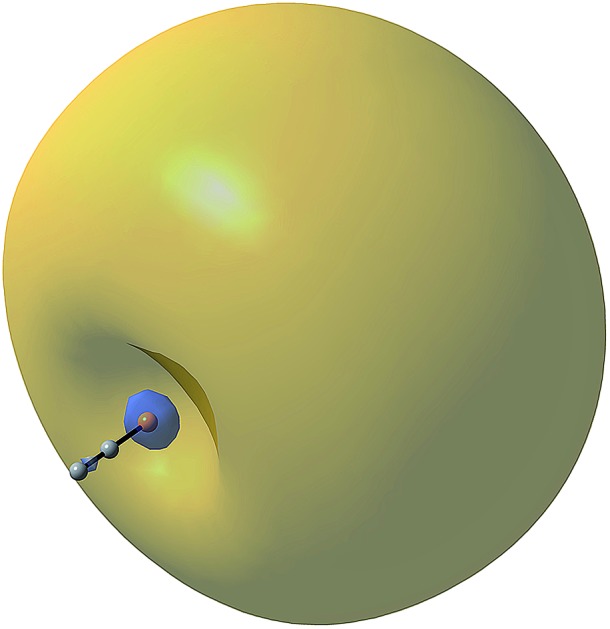
The spin–orbit coupling of a dipole-bound electron with the neutral core has been studied for the first time.

## Introduction

Polar molecules with large enough dipole moments can bind an electron in a diffuse orbital through charge–dipole interactions. Fermi and Teller first predicted a critical moment of 1.625 D for a stationary dipole to bind a charge.^1^ Further theoretical and experimental studies found that a minimum dipole moment of ∼2.5 D is required for a molecule to form a dipole-bound state (DBS).^2^–^8^ DBSs have been suggested to play an important role as a gateway for anion formation.^9^–^12^ DBSs can be formed from neutral molecules by Rydberg electron transfer^5^,^13^ and have been investigated using photoelectron spectroscopy (PES).^14^,^15^ Stable anions can have dipole-bound excited states near the electron detachment threshold if the neutral cores have large enough dipole moments.^16^,^17^ Dipole-bound excited states have allowed autodetachment spectroscopy^18^–^24^ and recently resonantly enhanced PES *via* vibrational autodetachment,^25^ which can yield highly non-Franck-Condon PE spectra and a wealth of spectroscopic information.^26^–^29^ The electron in a DBS resides in a diffuse orbital far from the neutral core, usually with a very small binding energy on the order of a few to a few tens of meV. The dipole-bound electron is known to have little effect on the structure of the neutral core. However, it is still an open question whether the electron in the diffuse dipole-bound orbital spin-couples with the electrons in the molecular core.

Dipole-bound states can be viewed as the analogues of Rydberg states in neutral molecules. However, it is well known that a Rydberg electron couples strongly with its cation core, particularly in low-*n* Rydberg states.^30^ For example, the Rydberg states of hydrogen halide molecules with a ^2^Π cationic state have been studied extensively using multi-photon ionization.^31^–^36^ All Hund's cases (a–e) have been considered in these studies to explain the experimental observations, highlighting the importance of the couplings between the Rydberg electron and the cation core. Despite the fact that both the dipole-bound and Rydberg electrons have little effect on the structures of the corresponding molecular cores, they do have major differences. Rydberg states are bound by the –1/*r* coulombic potential, while DBSs are bound by the –1/*r*^2^ charge–dipole potential. Hence, there are infinite Rydberg states in principle, but there is typically only one bound DBS. The weakly bound nature of the DBS raises an interesting question about the coupling of the dipole-bound electron and the electrons in the neutral core or lack thereof. However, to the best of our knowledge, this question has not been addressed.

The dicarbon–phosphorus cluster anion (C_2_P^–^) is an ideal candidate to probe the coupling of a dipole-bound electron with the neutral core. Previous spectroscopic studies showed that neutral C_2_P is an open-shell system with a valence electron configuration of 4σ^2^5σ^2^6σ^2^2π^4^7σ^2^3π^1^ and a ^2^Π ground state.^37^ Spin–orbit coupling splits the ground state into ^2^Π_1/2_ and ^2^Π_3/2_ with the ^2^Π_1/2_ spin–orbit state being lower in energy. The dipole moment of C_2_P was calculated to be 3.241 D,^38^ which is large enough to support a DBS. We have recently reported the PE spectra of C_2_P^–^ and measured the electron affinity (EA) of C_2_P to be 2.6328 ± 0.0006 eV.^39^ The valence electron configuration of the C_2_P^–^ anion is 4σ^2^5σ^2^6σ^2^2π^4^7σ^2^3π^2^ with the ^3^Σ^+^ electronic ground state.^39^ Hence, C_2_P^–^ is expected to possess a dipole-bound excited state with an excitation energy slightly below 2.6328 eV by promoting an electron from the 3π orbital to a dipole-bound orbital. We have indeed found such a diffuse σ-like orbital computationally, as shown in the ESI (Fig. S1†). If the dipole-bound electron couples with the open-shell C_2_P core, the DBS could be either in a^1^Π state or a^3^Π state, based on the 4σ^2^5σ^2^6σ^2^2π^4^7σ^2^3π^1^(*σ*_DBS_)^1^ configuration. The ^3^Π state would split into three spin–orbit states (^3^Π_0_, ^3^Π_1_, ^3^Π_2_), whereas the ^1^Π state is spin-forbidden and would be inaccessible in a single-photon excitation. However, if the dipole-bound electron does not couple with the neutral core, then there should be only two DBSs, which can be denoted as (^2^Π_1/2_)* and (^2^Π_3/2_)*, corresponding to the two spin–orbit states of neutral C_2_P. The purpose of this study is to find the DBSs in C_2_P^–^ using photodetachment spectroscopy and probe the nature of these diffuse excited states by resonant PES *via* vibrational autodetachment.^25^–^29^

## Results and discussion

**Fig. 1:**
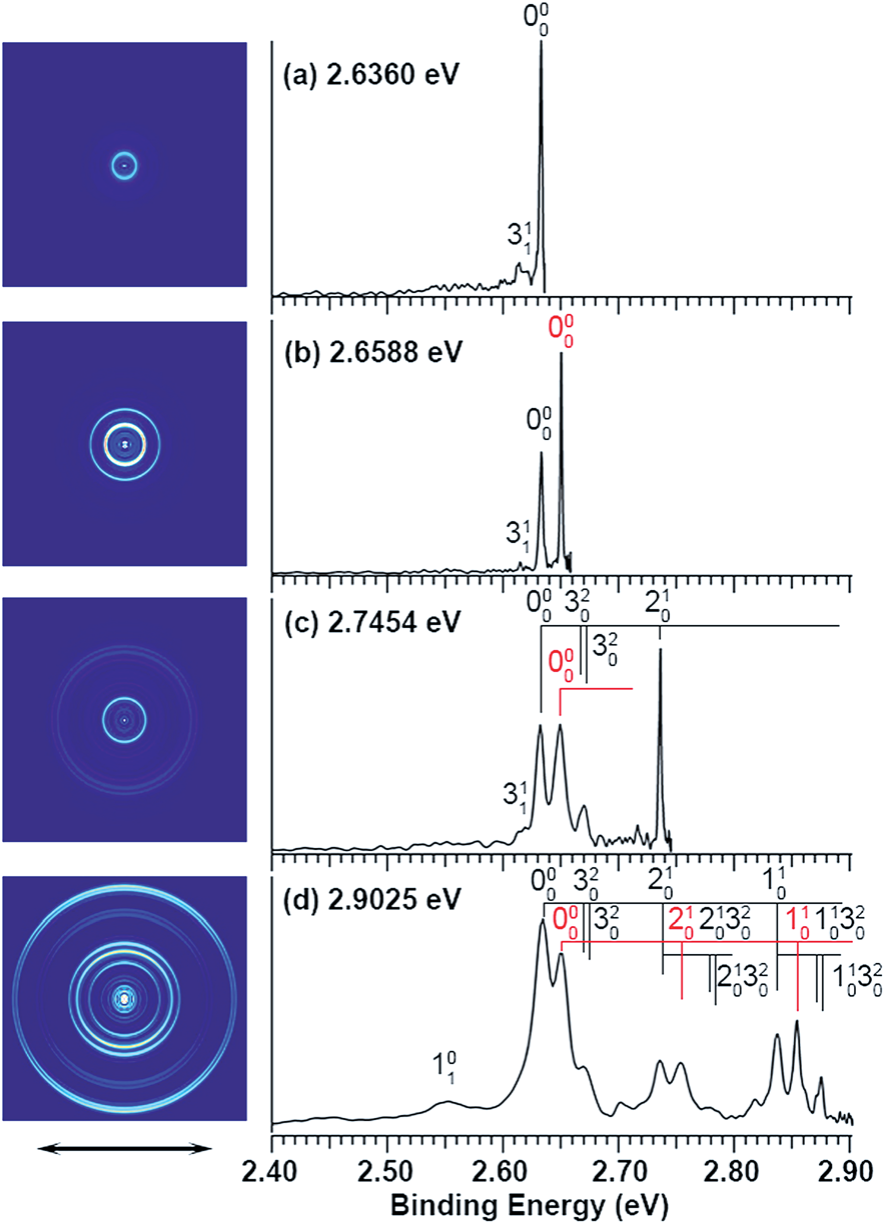
Non-resonant photoelectron spectra of C_2_P^–^ at four different photon energies. The spectrum at 2.9025 eV is from ref. 39 and is presented for comparison. The spectra in (a), (b), and (c) were recorded at photon energies corresponding to the short arrows in the photodetachment spectrum shown in Fig. 3.

The experiments were carried out using a high-resolution PE imaging system equipped with a laser vaporization cluster source.^40^ More experimental details are provided in the ESI.† The non-resonant PE spectra of C_2_P^–^ have been discussed in detail previously^39^ and they serve as the reference for the resonant PES in the current study. Additional non-resonant PE spectra have been measured in the current work at 2.6360, 2.6588, and 2.7454 eV, as compared with the 2.9025 eV spectrum reported previously in Fig. 1.

**Fig. 2:**
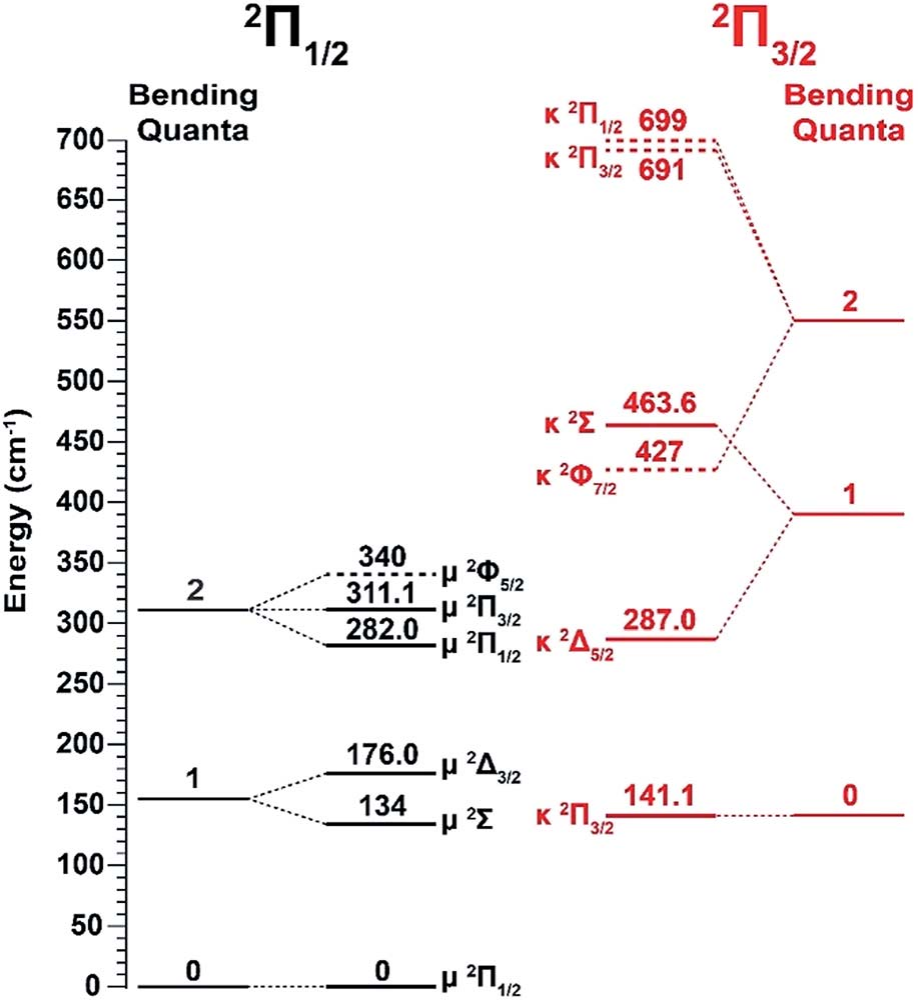
Renner–Teller splitting of the first three bending levels of C_2_P with the vibronic symmetries and observed energy levels according to ref. 37. The dashed lines are the calculated energy levels from ref. 37.

Vibrational features for the two spin–orbit states of C_2_P are denoted by black (^2^Π_1/2_) and red (^2^Π_3/2_) colors in Fig. 1. All three vibrational modes were observed for each spin–orbit state, *i.e.*, the C–C stretching (*ν*_1_), C–P stretching (*ν*_2_), and the bending mode (*ν*_3_).‡
‡The vibrational modes were labeled inadvertently according to decreasing frequencies (1 for C–C stretching, 2 for C–P stretching, and 3 for bending) in ref. 39. This labeling scheme is used in the current article for the PE spectra in Fig. 1 and 4 for consistency. It should be noted that this was different from the convention for triatomic molecules where the bending mode is always labeled as mode 2, as done in ref. 37. For the bending mode, only levels with even quanta (320) were observed. It should be pointed out that the Renner–Teller effect splits the bending levels in each spin–orbit state of C_2_P, which have been analyzed in detail previously.^37^ The vibronic levels, their symmetries, and known energies for the first three bending quanta are given in Fig. 2, according to ref. 37. The more intense 000 peak for the ^2^Π_3/2_ state in Fig. 1b and the 210 peak in Fig. 1c are due to a threshold enhancement effect, while the spectrum in Fig. 1d at 2.9025 eV photon energy represents the normal Franck–Condon transitions. The 000 peak for the ^2^Π_1/2_ state yielded a detachment threshold of 2.6328 ± 0.0006 eV, which defined the EA of neutral C_2_P.^39^ Features below the threshold are due to vibrational hot-bands from the anions. The same vibrational features for the two spin–orbit states are characterized by similar relative intensities in the non-resonant PE spectra, except for the threshold enhancement.

**Fig. 3:**
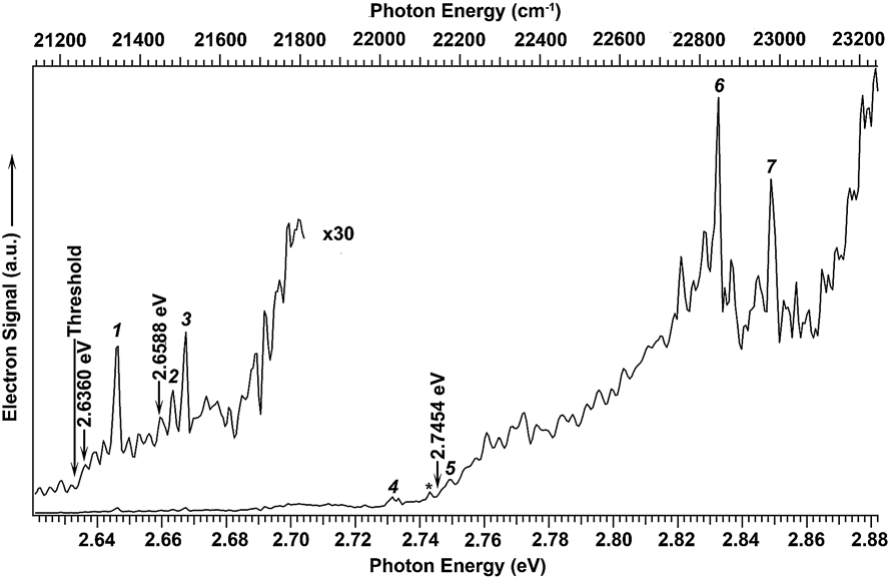
Photodetachment spectrum of C_2_P^–^ obtained by measuring the total electron yield as a function of photon energy. The electron detachment threshold at 2.6328 eV (ref. 39) is denoted by the long arrow. The short arrows indicate the photon energies used for the non-resonant photoelectron spectra presented in Fig. 1. The numbers (1–7) and the asterisk indicate resonant vibrational autodetachment peaks from dipole-bound excited states of C_2_P^–^.

We searched for the DBS of C_2_P^–^ experimentally using photodetachment spectroscopy by measuring the total electron yield, while scanning the detachment laser from 2.62 to 2.88 eV, as shown in Fig. 3. Electron signals appeared promptly at the detachment threshold of 2.6328 eV (indicated by a long arrow). The photodetachment cross section was expected to smoothly increase with the photon energy and exhibit steps as new detachment channels (electronic or vibrational) opened up. In addition, several resonant peaks were observed and marked with numbers 1–7 or an asterisk in Fig. 3. These peaks indicated the presence of a dipole-bound excited state, representing autodetachment from vibrationally excited levels of the DBS of C_2_P^–^. No other resonant peaks were observed in this energy range; the spikes in the figure were due to the relatively poor signal-to-noise ratios as a result of the weak and sometimes unstable C_2_P^–^ mass signals. The excitation energies of the observed resonant peaks and their assignments are given in Table 1. The ground vibrational level of the DBS should be just below the detachment threshold and could only be observed *via* resonant two-photon detachment, which was usually very weak.^25^–^29^ The overall weak signals for the C_2_P^–^ anions prevented us from observing the resonant two-photon transition. However, the ground vibrational state of the DBS can be deduced from resonant PE spectra (*vide infra*).

**Table 1:** Photon energies (in nm, eV, and cm^–1^) and assignments of the observed resonant peaks in the photodetachment spectrum of C_2_P^–^ shown in Fig. 2

Peak	nm	eV	cm^–1^	Assignment	Shift^*a*^ (cm^–1^)	Vibrational level^*b*^ (cm^–1^)	BE^*c*^ (cm^–1^)
1	468.82	2.6446	21 330	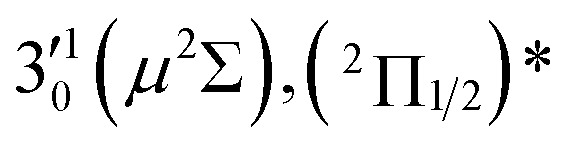	95	134	39
2	465.42	2.6639	21 486	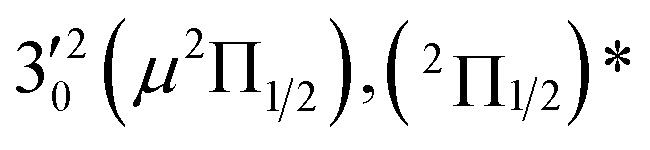	251	282.0	31
				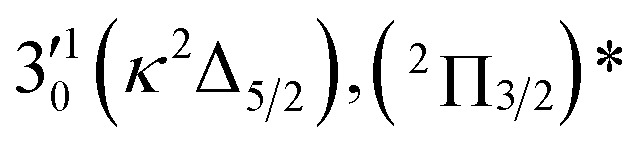	110	145.9	36
3	465.02	2.6662	21 504	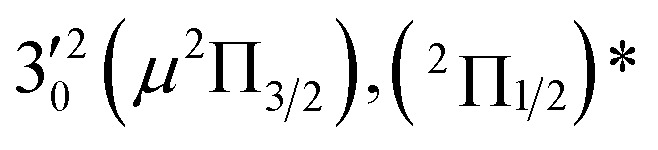	269	311.1	42
4	453.91	2.7315	22 031	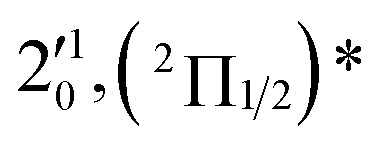	796	834.8	39
5	451.02	2.7490	22 172	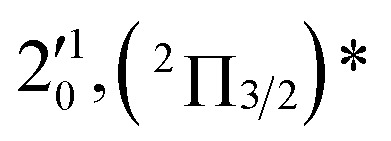	796	840.4	44
6	437.70	2.8326	22 846	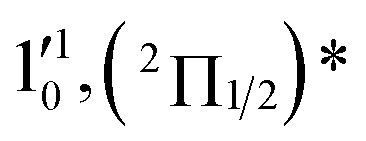	1611	1644.3	33
7	435.03	2.8500	22 987	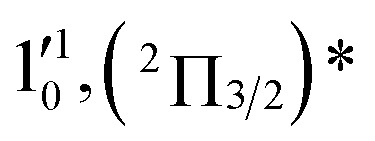	1611	1644.2	33
*	452.00	2.7430	22 124	—	889	—	—

^*a*^The shift is calculated as the difference between the photon energy of the resonant peak and the detachment threshold of the corresponding neutral spin–orbit states.

^*b*^Vibrational energy levels of the corresponding neutral C_2_P states from ref. 37. See also Fig. 5.

^*c*^The deduced binding energy of the dipole-bound electron, calculated as the difference between the neutral vibrational level and the corresponding shift. The vibrational frequencies and levels of the dipole-bound states are assumed to be the same as those of neutral C_2_P. The average binding energy is computed to be 37 ± 6 cm^–1^.

**Fig. 4:**
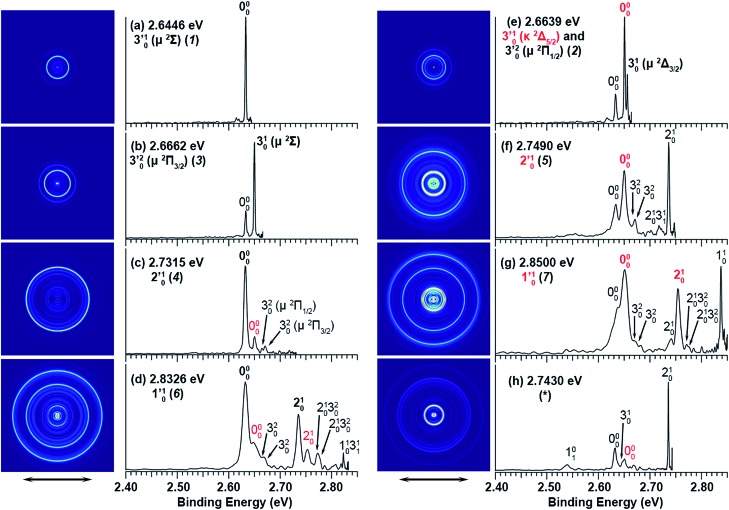
Resonant photoelectron spectra of C_2_P^–^ at (a) 2.6446 eV (468.82 nm), (b) 2.6662 eV (465.02 nm), (c) 2.7315 eV (453.91 nm), (d) 2.8326 eV (437.70 nm), (e) 2.6639 eV (465.42 nm), (f) 2.7490 eV (451.02 nm), (g) 2.8500 eV (435.03 nm), and (h) 2.7430 eV (452.01 nm). The black labels correspond to the ^2^Π_1/2_ spin–orbit state of C_2_P and the red labels correspond to the ^2^Π_3/2_ spin–orbit state. The resonant peak numbers (1–7) from Fig. 3 and the vibrational levels of the dipole-bound excited states are given in the resonant spectra. The resonantly enhanced vibrational peaks are labeled in boldface. The double arrows below the images indicate the directions of the laser polarization.

The three short arrows in Fig. 3 indicate the photon energies used to obtain the non-resonant PE spectra presented in Fig. 1. Resonantly enhanced PE spectra were recorded at the photon energies corresponding to peaks 1–7 and *, as shown in Fig. 4, where the non-resonant spectrum at 2.9025 eV is also given for comparison in Fig. 1 to show the normal Franck–Condon intensities. The resonant peaks in Fig. 3 correspond to excitations to vibrational levels of the DBS, followed by autodetachment, which obeys the Δ*v* = –1 propensity rule due to the similarity of the neutral core of the DBS and the neutral final state.^41^,^42^ Hence, the resonant PE spectra are highly non-Franck–Condon and one or more peaks are enhanced in each spectrum, compared to the non-resonant PE spectrum (Fig. 1d). The resonantly enhanced peaks are labeled in boldface in Fig. 4; the vibrational levels for the ^2^Π_1/2_ spin–orbit state are labeled in black and those for the ^2^Π_3/2_ state are given in red. In addition to the photon energy used, the resonant peak number from Fig. 3 and the DBS vibrational level (indicated by an apostrophe ') are also given in Fig. 4. The 000 transition of the ^2^Π_1/2_ state is enhanced in Fig. 4a and c, while the 000 and 210 transitions of the ^2^Π_1/2_ state are enhanced in Fig. 4d. The 000 transition of the ^2^Π_3/2_ state is enhanced in Fig. 4e and f. In addition, a new peak assigned to the 310(*μ*^2^Δ_3/2_) vibronic level of the ^2^Π_1/2_ state (see Fig. 2) is observed in Fig. 4e. In Fig. 4f, the intense 210 peak of the ^2^Π_1/2_ state is due to threshold enhancement similar to that observed in Fig. 1c. The 000 and 210 levels of the ^2^Π_3/2_ state are resonantly enhanced in Fig. 4g, where the intense 110 peak of the ^2^Π_1/2_ state is again due to threshold enhancement. Finally, the enhanced peak in Fig. 4b (134 cm^–1^ above the 000 peak) is due to the 310(*μ*^2^Σ) vibronic level of the ^2^Π_1/2_ state, and is very close to the 000 peak of the ^2^Π_3/2_ state (Fig. 2).

To understand the spectral assignments and the resonant PE spectra, we need to consider the electronic selection rule for autodetachment, in addition to the Δ*v* = –1 vibrational propensity rule. For the linear C_2_P^–^ molecule, electron detachment should follow the Δ*J* = ±1/2 selection rule. If the dipole-bound electron in C_2_P^–^ couples with the core electrons, the DBS should be a ^3^Π_*J*_ state with three spin–orbit components, *J* = 0, 1, 2. Hence, we would expect the ^3^Π_0_ spin–orbit DBS to autodetach only to the ^2^Π_1/2_ state of C_2_P, the ^3^Π_2_ DBS to the ^2^Π_3/2_ state only, and the ^3^Π_1_ DBS to both the ^2^Π_1/2_ and ^2^Π_3/2_ states of C_2_P. However, if the dipole-bound electron in C_2_P^–^ is not coupled with the electrons in the C_2_P core, we would expect to have only two DBSs, which can be denoted as (^2^Π_1/2_)* and (^2^Π_3/2_)*, corresponding to the two neutral spin–orbit states, respectively. Each of the DBSs would autodetach only to the corresponding neutral spin–orbit state: (^2^Π_1/2_)* → ^2^Π_1/2_ + e^–^ and (^2^Π_3/2_)* → ^2^Π_3/2_ + e^–^. The observed resonant PE spectra shown in Fig. 4 are consistent with the latter, *i.e.*, there are only two DBSs in C_2_P^–^. In each resonant PE spectrum, only the vibrational levels of one spin–orbit component are enhanced. We did not observe any resonances in the photodetachment spectrum that led to simultaneous enhancement of the same vibrational levels of the two spin–orbit states.

**Fig. 5:**
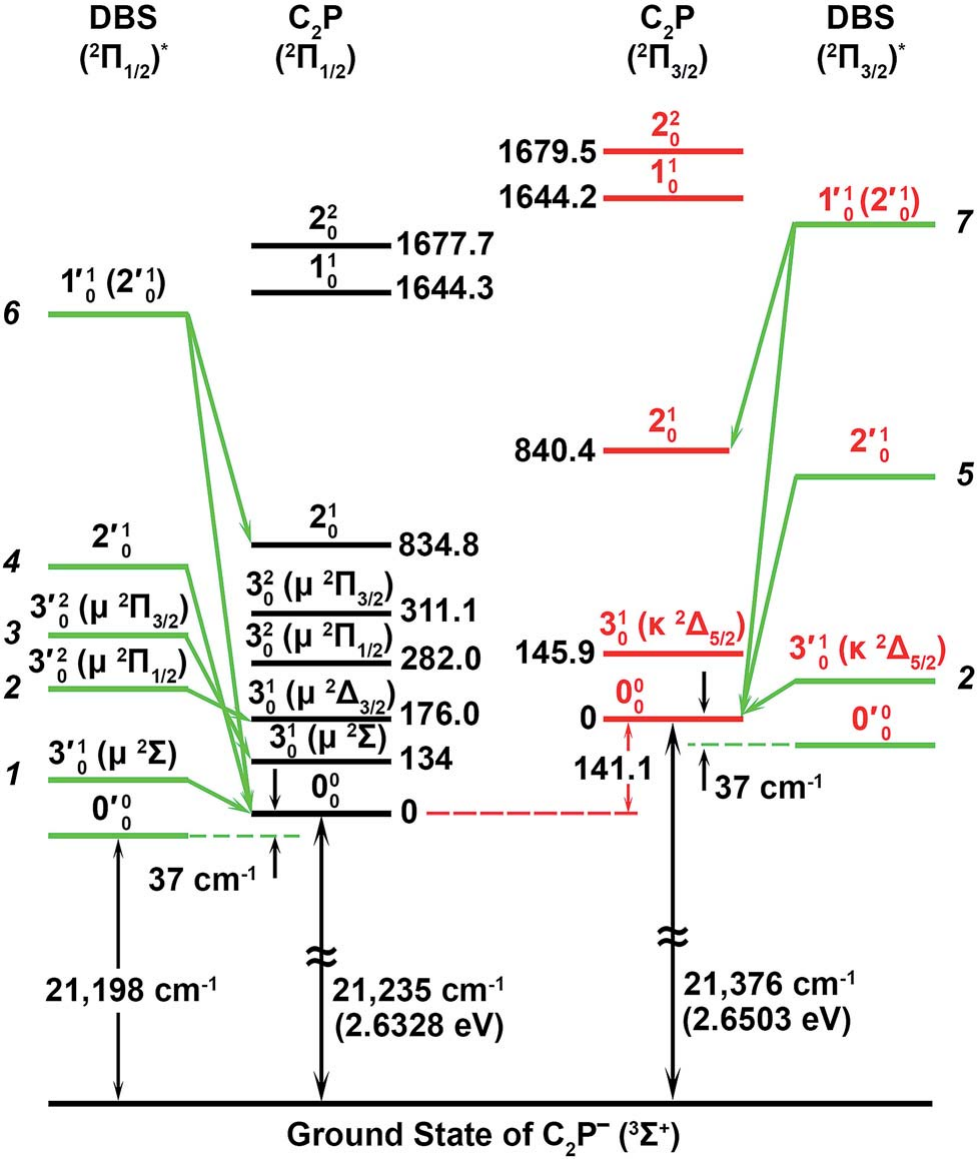
A schematic energy level diagram showing the vibrational levels of the observed two dipole-bound excited states of C_2_P^–^ and their autodetachment to the two spin–orbit states of neutral C_2_P. The detachment thresholds of the ^2^Π_1/2_ ground spin–orbit state of C_2_P and the ^2^Π_3/2_ excited state are given, as well as the deduced binding energy (37 ± 6 cm^–1^) of the DBSs. The vibrational levels and energies for neutral C_2_P are from ref. 37 and 39. The vibronic symmetries of the bending levels are given in parentheses.

In addition to the Δ*v* = –1 propensity rule, vibrational autodetachment is also mode-selective,^25^–^29^ *i.e.*, a vibrational level 
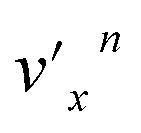
 of mode 
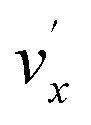
 of the DBS preferentially autodetaches to the *v*_*x*_^*n*–1^ vibrational level of the same mode of the neutral species (the apostrophe ' is used to indicate the same vibrational mode for the DBS). As shown recently, the vibrational frequencies of the DBS are the same as those of the neutral species.^25^–^29^ Using the vibrational propensity rule and the known vibrational levels of neutral C_2_P (Fig. 2 and 5, and Table 1), we can understand the resonant PE spectra, assign the resonant peaks in Fig. 3, and deduce the DBS vibrational ground state.

The 000 peak of the ^2^Π_1/2_ spin–orbit state of C_2_P is enhanced in the resonant PE spectra in Fig. 4a, c and d, which were recorded at photon energies corresponding to resonant peaks 1 (2.6446 eV), 4 (2.7315 eV), and 6 (2.8326 eV), respectively. These resonant peaks should correspond to fundamental vibrational excitations in the (^2^Π_1/2_)* DBS to obey the Δ*v* = –1 propensity rule. The excitation energies of resonant peaks 1, 4, and 6 are 0.0118 eV (95 cm^–1^), 0.0987 eV (796 cm^–1^), and 0.1998 eV (1611 cm^–1^) above the detachment threshold of 2.6328 eV, respectively. As shown in Fig. 5, peaks 1, 4, and 6 should correspond to the 
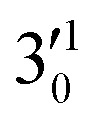
 (*μ*^2^Σ), 
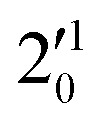
, and 
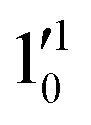
 vibrational levels of the (^2^Π_1/2_)* DBS, respectively, which autodetach to the 000 level of the ^2^Π_1/2_ spin–orbit state of C_2_P *via* transfer of one vibrational quantum to the dipole-bound electron. Since the vibrational frequencies of the DBS are the same as those of the corresponding neutral states, peaks 1, 4, and 6 indicate a vibrational ground state for the (^2^Π_1/2_)* DBS, which is on average 37 cm^–1^ (±6 cm^–1^) below the detachment threshold of the ^2^Π_1/2_ state of C_2_P, *i.e.*, the binding energy of the DBS. There also appears to be some enhancement of the 210 level in Fig. 4d and this is likely due to the proximity of the 
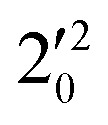
 (1677.7 cm^–1^) and 
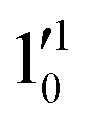
 (1644.3 cm^–1^) vibrational levels (Fig. 5).

The 000 peak of the ^2^Π_3/2_ spin–orbit state of C_2_P is enhanced in the resonant PE spectra in Fig. 4e–g, which were recorded at photon energies corresponding to resonant peaks 2 (2.6639 eV), 5 (2.7490 eV), and 7 (2.8550 eV), respectively. These resonant peaks should correspond to fundamental vibrational excitations in the (^2^Π_3/2_)* DBS to obey the Δ*v* = –1 propensity rule. The excitation energies of the resonant peaks 2, 5, and 7 are 0.0136 eV (110 cm^–1^), 0.0987 eV (796 cm^–1^), and 0.1997 eV (1611 cm^–1^) above the detachment threshold of 2.6503 eV for the ^2^Π_3/2_ spin–orbit state of C_2_P, respectively. As shown in Fig. 5, peaks 2, 5, and 7 should correspond to the 
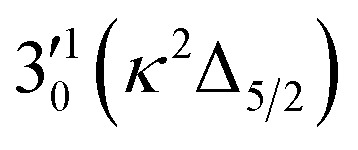
, 
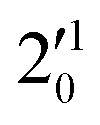
, and 
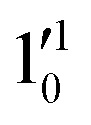
 vibrational levels of the (^2^Π_3/2_)* DBS, respectively, which autodetach to the 000 level of the ^2^Π_3/2_ spin–orbit state of C_2_P. The vibrational ground state of the (^2^Π_3/2_)* DBS is also deduced on average to be 37 cm^–1^ (±6 cm^–1^) below the ^2^Π_3/2_ state of C_2_P (Fig. 5). Again, the enhancement of the 210 level in Fig. 4g is likely due to the proximity of the 
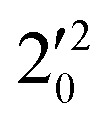
 and 
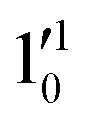
 vibrational levels (Fig. 5).

The spectrum in Fig. 4b was recorded at the photon energy of resonant peak 3 (2.6662 eV). The 310(*μ*^2^Σ) vibronic level of the ^2^Π_1/2_ spin–orbit state of C_2_P was enhanced, suggesting that the autodetaching state was from the 3′_0_^2^(*μ*^2^Π_3/2_) vibronic level of the (^2^Π_1/2_)* DBS (Fig. 5), in agreement with the dipole selection rule of the vibronic transition. In Fig. 4e, the 310(*μ*^2^Δ_3/2_) vibronic peak of the ^2^Π_1/2_ spin–orbit state of C_2_P is also enhanced, in addition to the enhancement of the 000 peak of the ^2^Π_3/2_ spin–orbit state. This was because of the near degeneracy of the 
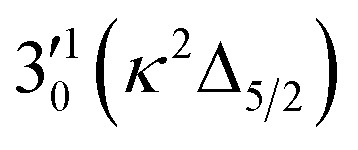
 vibronic level (287.0 cm^–1^) of the (^2^Π_3/2_)* DBS and the 
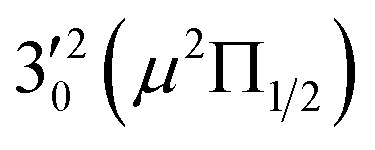
 vibronic level (282.0 cm^–1^) of the (^2^Π_1/2_)* DBS (Fig. 2). Dipole selection rules allow for only one autodetachment transition from the 
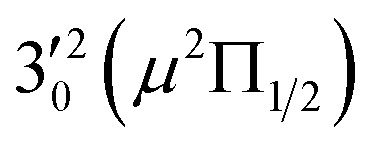
 resonant state to the 310(*μ*^2^Δ_3/2_) neutral vibronic state (Fig. 5), which is observed to be 176 cm^–1^ above the ground vibrational level of the ^2^Π_1/2_ spin–orbit state of C_2_P, in good agreement with the previous measurement.^37^

Finally, the resonant PE spectrum corresponding to the resonant peak labeled as * (2.7430 eV) in Fig. 3 is shown in Fig. 4h. The intense 210 peak is likely due to threshold enhancement, similar to that shown in Fig. 1c. Clearly, the 000 peak and possibly the 310 peak of the ^2^Π_1/2_ spin–orbit state of C_2_P are enhanced, suggesting the excitation to a vibrational level of the (^2^Π_1/2_)* DBS. The resonant energy at 2.7430 eV is 0.1102 eV (889 cm^–1^) above the EA of C_2_P, indicating a DBS vibrational level at 926 cm^–1^ (889 + 37 cm^–1^). This could correspond to a higher bending vibronic level of the (^2^Π_1/2_)* DBS, according to ref. 37. However, we cannot assign this resonance definitively without precise knowledge of the vibronic levels of C_2_P at around 926 cm^–1^. The angular distributions of all the observed peaks in the resonant and non-resonant PE spectra are shown in Fig. S2.† The nearly isotropic distributions of the resonantly enhanced peaks provide further confirmation of our assignments because the lifetime of a DBS is typically much longer than the rotational period of a molecule.^43^–^45^

## Conclusions

It is now clear that there are only two dipole-bound excited states in C_2_P^–^, (^2^Π_1/2_)* and (^2^Π_3/2_)*, each autodetaching only to a single spin–orbit state of neutral C_2_P (^2^Π). The binding energy of each DBS is 37 ± 6 cm^–1^. This observation suggests that the spin of the dipole-bound electron is not coupled with the electrons in the neutral C_2_P core. Hence, the electron in the dipole-bound excited states of C_2_P^–^ can be viewed as a quasi-free electron. This conclusion raises some interesting questions. Does the uncoupling between the dipole-bound electron and the electrons in the molecular core depend on the binding energy of the DBS, *i.e.*, the magnitude of the dipole moment, or is it universal for DBSs? Does this observation mean that the electron spin is not conserved during excitation from the anion ground state to the dipole-bound excited states? The current study further demonstrates that resonant PES *via* vibrational autodetachment is a powerful technique to probe the nature of dipole-bound excited states.

## Conflicts of interest

There are no conflicts to declare.

## Supplementary Material

Supplementary information
