# Supplementary material for: Probing the coupling of a dipole-bound electron with a molecular core
Source: Chem Sci. 2018 Nov 15;10(5):1386–91. doi: 10.1039/c8sc04771e (PMC6354839; doi:10.1039/c8sc04771e)
Supplement: Supplementary file 1 [file SC-010-C8SC04771E-s001.pdf]

Electronic Supplementary Material (ESI) for Chemical Science.
This journal is © The Royal Society of Chemistry 2018

Supporting Information for

Probing the Coupling of A Dipole-Bound Electron with the Molecular Core

Joseph Czekner, Ling Fung Cheung, G. Stephen Kocheril, and Lai-Sheng Wang*

Brown University, Department of Chemistry, 324 Brook Street, Providence, RI 02912, USA

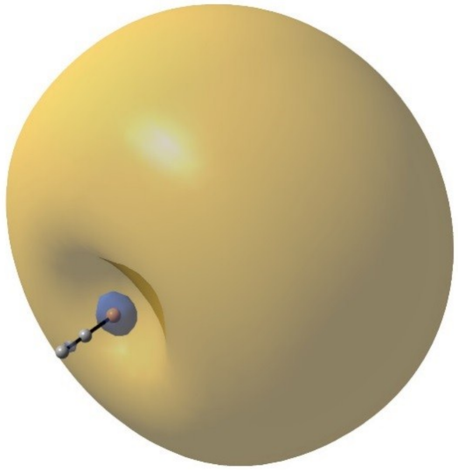

Figure S1. The calculated wave function of the dipole-bound excited state of C_2P^-, based on theoretical calculations using the ωB97XD functional[S1] and the daug-cc-pVTZ basis set[S2] augmented with an additional *s* and *p* function in Gaussian 09.[S3]

Experimental Details

The C_2P^- anions were produced by laser ablation of a disk target composed of graphite, red phosphorus, and bismuth (served both as a binder and a source of Bi^- for spectral calibration). The laser-induced plasma was cooled by a He carrier gas containing 10% Ar at a backing pressure of 10 atm to initiate nucleation. The nascent clusters were entrained in the carrier gas and underwent a supersonic expansion. The negatively-charged clusters were extracted from the cluster beam and analyzed by time-of-flight mass spectrometry. Mixed C-P clusters with different stoichiometries were observed. The C_2P^- anions of current interest were mass selected and photodetached using a Deyang dye laser system in the interaction zone of a multi-lens imaging system.[S4] Photoelectrons were extracted and projected onto a pair of 75-mm diameter micro-channel plates coupled to a phosphor screen and recorded using a charge-coupled device camera. The PE images were analyzed using the maximum entropy method (MEVELER).[S5] The PE spectra were calibrated using the known detachment energies of Au^- and Bi^- with various photon energies; the laser wavelengths were calibrated with a Bristol 821 wavelength meter. Typical kinetic energy (KE) resolution of the imaging lens is 1.2 cm^{-1} for low KE electrons (5.1 cm^{-1}) and ~0.6% ΔKE/KE for high KE electrons (above 2 eV).[S4]

Photoelectron Angular distributions (PADs)

Angular distributions of photoelectrons are useful to analyze the angular momentum of the detached photoelectron. PADs can be quantified using the anisotropy parameter, β, as shown in Eq. 1:

$$d\sigma/d\Omega = \sigma_{Tot}/4\pi[1 + \beta P_2(\cos\theta)] \quad (1)$$

Here σ_{Tot} is the total cross section, P_2 is the second order Legendre polynomial, and θ is the angle of the outgoing electron relative to the laser polarization.[S6] The PAD can be approximated by

$$I(\theta) \sim [1 + \beta P_2(\cos\theta)] \quad (2)$$

where β can have any value between -1 and 2. This model works reasonably well for single-photon detachment from randomly oriented particles. Therefore, if an electron is detached from an atomic *s*-orbital (l = 0) the outgoing electron will have one unit of angular momentum (l = 1) and the resulting β value will be 2. Because molecular orbitals can be approximated as a linear

combination of atomic orbitals, interpreting β for a molecular system is not a trivial process.[S7] However, we can use the angular distributions to differentiate between resonant and non-resonant photoelectron spectra, particularly at higher electron kinetic energies (above 0.05 eV) where threshold effects are less noticeable. Resonantly-enhanced features are found to be nearly isotropic with β ~ 0. The angular distributions of the main features in Fig. 3, Fig. S2, and Fig. S3 have been analyzed and the beta values are shown in Fig. S4.

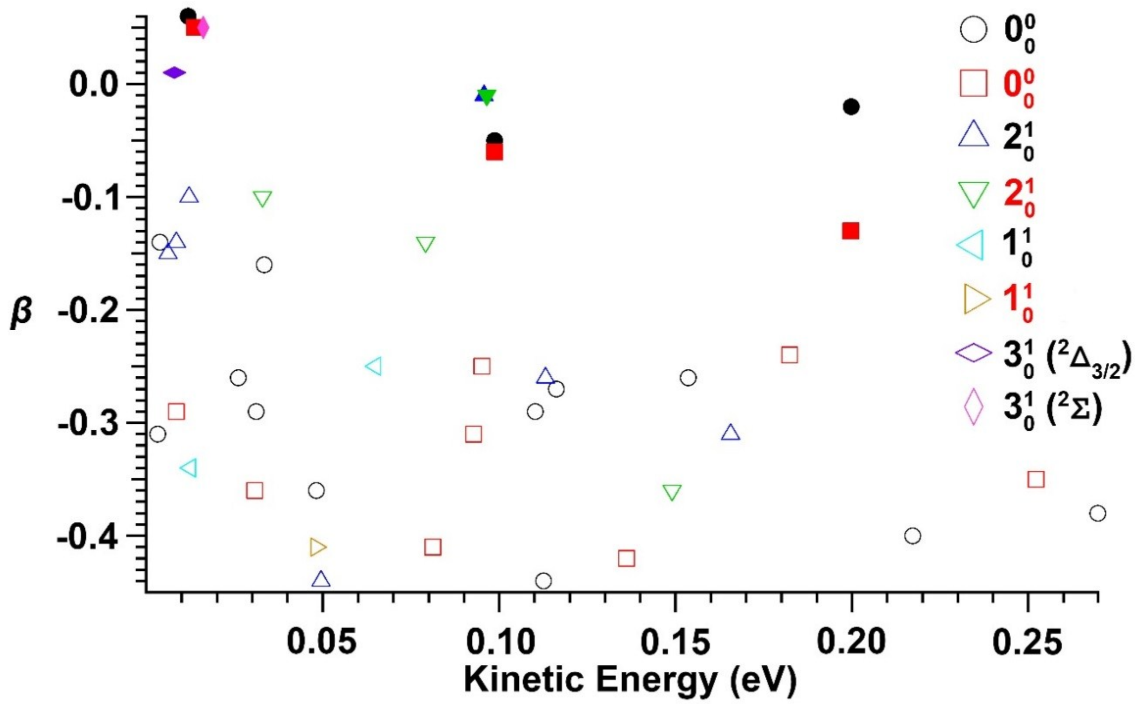

Figure S2. Beta values of the main vibrational features in the PE spectra in Fig. 1 and Fig. 4. Solid shapes denote enhanced peaks in the dipole-bound resonant spectra, which are all nearly isotropic (β ~ 0).

References

S1. D. E. Woon and T. H. Dunning Jr., *J. Chem. Phys.*, 1994, **100**, 2975-2988.

S2. J. D. Chai and M. Head-Gordon, *Phys. Chem. Chem. Phys.* 2008, **10**, 6615-6620.

S3. M. J. Frisch, G. W. Trucks, H. B. Schlegel, G. E. Scuseria, M. A. Robb, R. J. Cheeseman, G. Scalmani, V. Barone, G. A. Petersson, H. Nakatsugi, X. Li, M. Caricato, A. Marenich, J. Bloino, B. G. Janesko, R. Gomperts, B. Mennucci, H. P. Hratchian, J. V. Ortiz, A. F. Izmaylov, J. L. Sonnenberg, D. Williams-Young, F. Ding, F. Lipparini, F. Egidi, J. Goings, B. Peng, A. Petrone, T. Henderson, D. Ranasinghe, V. G. Zakrzewski, J. Gao, N. Rega, G. Zheng, W. Liang, M. Hada, M. Ehara, K. Toyota, R. Fukuga, J. Hasegawa, M. Ishida, T. Nakajima, Y. Honda, O. Kitao, H. Nakai, T. Vreven, K. Throssell, J. A. Montgomery, Jr., J. E. Peralta, F. Ogliaro, M. Bearpark, J. J. Heyd, E. Brothers, K. N. Kudin, V. N. Staroverov, T. Keith, R. Kobayashi, J. Normand, K. Raghavachari, A. Rendell, J. C. Burant, S. S. Iyengar, J. Tomasi, M. Cossi, J. M. Millam, M. Klene, C. Adamo, R. Cammi, J. W. Ochterski, R. L. Martin, K. Morokuma, O. Farkas, J. B. Foresman, D. J. Fox, *Gaussian 09, Revision C.01;* Gaussian Inc.: Wallingford CT, 2016.

S4. I. Léon, Z. Yang, H. T. Liu, and L. S. Wang, *Rev. Sci. Instrum.* 2014, **85**, 083106.

S5. B. Dick, *Phys. Chem. Chem. Phys.* 2014, **16**, 570-580.

S6. J. Cooper and R. N. Zare, *J. Chem. Phys.* 1968, **48**, 942-943.

S7. A. Sanov and R. Mabbs, *Int. Rev. Phys. Chem.* 2008, **27**, 53-85.
